# Liposomal Nanocarriers to Enhance Skin Delivery of Chemotherapeutics in Cancer Therapy

**DOI:** 10.3390/bioengineering12020133

**Published:** 2025-01-30

**Authors:** Xiangli Liu, Robert A. Falconer

**Affiliations:** School of Pharmacy and Medical Sciences, Faculty of Life Sciences, University of Bradford, Bradford BD7 1DP, UK; r.a.falconer1@bradford.ac.uk

**Keywords:** skin delivery, cancer therapy, chemotherapeutics, liposomal nanocarriers, conventional liposomes, deformable liposomes

## Abstract

Cancer chemotherapeutics administered to cancer patients via traditional oral or parenteral routes often encounter poor bioavailability and severe systemic side effects. Skin delivery is a promising alternative route with reduced side effects and improved therapeutic efficacy and has gained significant attention in recent years. With conventional or deformable liposomal nanocarriers as a skin permeation strategy, cancer chemotherapeutics can be delivered via skin route, offering an option for more efficient therapy. This review summarizes the recent advances in liposome nanocarrier efficacy to enhance the skin delivery of chemotherapeutics with a wide range of physicochemical properties (log P_oct_ from −0.89 to 5.93, MW from 130 to 1415) in targeting local skin cancer, breast cancer, and tumor metastasis and delivering the drug to systemic circulation to treat distal cancers. The potential mechanisms of skin permeation enhancement by different type of liposomes are also discussed in this review.

## 1. Introduction

Cancer is the leading cause of death globally. Based on the latest WHO statistics, there were an estimated 20 million new cancer cases and 9.7 million deaths in 2022. Over 35 million new cancer cases are predicted in 2050, a 77% increase from the estimated 20 million cases in 2022. The rapidly growing global cancer burden has posed a great challenge to the health care professionals. The need to tackle the practical problems with anticancer therapy increased proportionately [1]. There are multiple treatment options available in clinics including surgical resection, chemotherapy [2,3,4,5], radiotherapy [6], biological therapy [7], and immunotherapy [8].

Currently, chemotherapeutic drugs are primarily administered to cancer patients via traditional oral and parenteral routes, which often encounter problems such as first-pass hepatic metabolism, poor bioavailability, severe systemic side effects, and patient incompliance [9]. Furthermore, only a small portion of drugs can reach the target site, and with reduced therapeutic effect. Multiple doses are required to maintain the effective drug concentration at the cancer site, which may cause drug resistance and severe side effect especially in the setting of long-term medication treatment [10,11]. The disadvantages of conventional delivery demand the development of alternative routes with reduced side effects and improved therapeutic efficacy. Skin delivery has gained significant attention in recent years due to a number of advantages and could be a promising route to overcome the disadvantages in cancer therapy [12,13]. It can prolong the therapeutic effect by controlling the rate of drug release, decrease the dose frequency, and reduce severity of toxic effects associated with oral or parenteral administration of drugs [14]. Liposomal nanocarrier systems have been explored and investigated as topical and transdermal delivery systems for cancer therapy, because they could enhance the penetration of therapeutics through the skin barrier, resulting in drug delivery close to the lesion to improve the local drug concentration and thus enhance the therapeutic effects [15,16]. The effectiveness of liposomal formulations has been demonstrated in the topical and transdermal delivery of drugs with different physicochemical properties [17,18]. This review will focus on characteristics of skin delivery and liposomal nanocarriers, as well as recent advances by employing liposomal nanocarriers to enhance the skin delivery of clinically approved chemotherapeutic drugs aiming for optimal anticancer therapy. In addition, the experimental parameters and drug physicochemical properties used in these studies are briefly summarized in Table 1.

## 2. Structure of the Skin and Skin Delivery Routes

Skin covers a surface area from 1.5 to 2.0 m^2^ and is regarded as the largest organ of the human body. From a pharmaceutical point of view, skin as a route of drug delivery offers advantages over other routes of administration. These include ease of use and withdrawal in the advent of side-effects, avoidance of first-pass metabolism, suitability for drugs with low oral bioavailability, improved patient compliance, and sustained release [19,20]. However, despite the described advantages, most drugs are not amenable to this mode of administration due to the barrier function of the skin. The skin consists of three principal and distinct layers: stratum corneum (SC) (10–20 μm thick), viable epidermis (50–100 μm thick), and dermis (1–2 mm thick) [21]. A fatty subcutaneous layer resides beneath the dermis.

**Figure 1:**
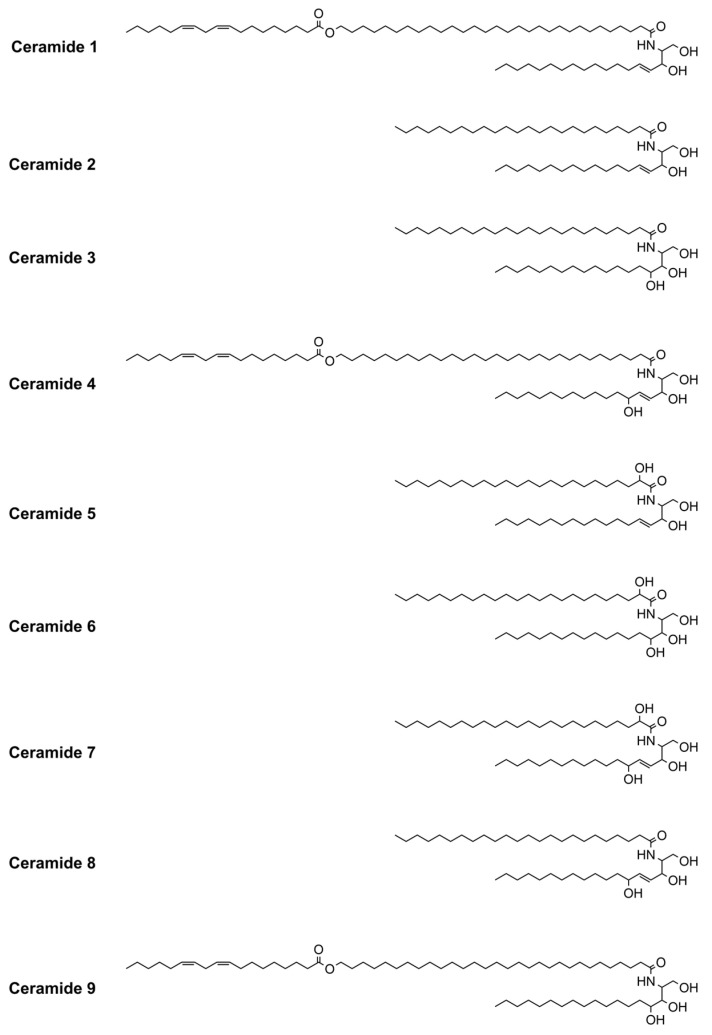
The chemical structure of ceramides.

The transdermal permeation rate of most drugs is limited by the SC [22,23]. The SC comprises 10–15 layers of flat keratin-filled cells, closely packed in a non-polar lipid matrix, mainly composed of ceramides (50%), cholesterol (25%), free fatty acids (10–15%), and sterol esters (5%) [24,25]. Ceramides are categorized into nine subgroups (Figure 1), whose headgroups can form lateral intermolecular hydrogen bonds [26]. Ceramides and other lipids adopt a highly ordered, three-dimensional structure of stacked densely packed lipid layers (lipid lamellae): the lateral and lamellar lipid organization in the intercellular space [27]. The extracellular lipid matrix of the stratum corneum comprises not only the structure that limits transdermal delivery of hydrophilic drugs, but also the so-called stratum corneum “reservoir” [28], within which lipophilic drugs can accumulate and be slowly released.

**Figure 2:**
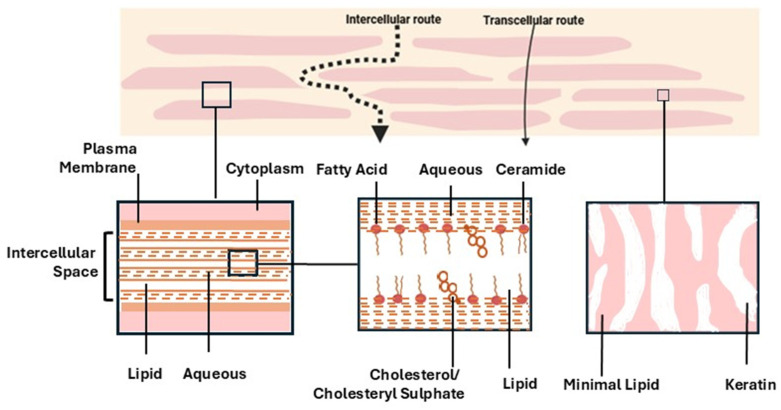
Simplified diagram of bricks-and-mortar model of stratum corneum and the primary transport pathway through SC: intercellular route. Modified from [27].

This SC structure has been described as the “bricks and mortar” model where the bricks are the corneocytes and the mortar refers to the multiple bilayers of lipids [29,30,31] (Figure 2). The thickness of the SC is different in each body part; for example, it is about 15 μm thick in the abdominal skin and 10 μm thick in the dorsal skin. The primary transport pathway for most drugs passively traversing the SC is the intercellular lipid region [32].

The viable epidermis layer has a higher degree of hydration, renders a faster diffusion rate [33]. The dermis layer contains blood capillaries, sebaceous glands, sweat glands, hair follicles, lymphatic vessels, and nerve endings. The capillary network constructs the microcirculation system of the skin for absorption of drugs into systemic circulation, and other functions such as nutrient supply and temperature regulation.

Drug permeation across the skin is a multistep process. For skin application, the drug partitions into the intercellular lipids of SC; it may reach different layers by diffusion depending on its physicochemical properties, whereby lipophilicity and molecular weight play vital roles [34]. Some drugs could penetrate the shallower layer of the skin, making it possible for topical treatment, which has potential for performing site-specific skin cancer and breast cancer therapy. For transdermal application, the pharmaceuticals need to have suitable physicochemical properties to diffuse through the viable epidermis and enter the dermis layer, where the drug enters the systemic circulation for distal cancer therapy.

It should be noted that hair follicles may act as a penetration pathway or potential reservoirs for topically applied compounds [35,36], although they account for only about 0.1% of the skin surface area [37].

Since the first commercial transdermal product (scopolamine) was introduced in the global market in 1981, about 20 molecules utilizing transdermal technology have been approved over the past four decades. The limited number reflects the difficulty of meeting the dual challenge of potent pharmacological activity and the correct physicochemical properties to enable skin penetration [38]. The ideal drug characteristics for transdermal drug delivery are low molecular weight (<500 Da), moderately lipophilic (log P_oct_: 1–3), and high pharmacological potency [38,39].

To overcome the challenge, physical and chemical strategies have been introduced to enhance drug permeation across skin [27,40]. Physical strategies use the techniques such as microneedles, iontophoresis, electroporation, and laser ablation to facilitate the drug bypassing the SC [41,42,43]. The chemical strategies employ penetration enhancers, nano delivery systems such as liposome, emulsion for assisting the drug penetration into the skin by modulating the barrier function of the SC [44,45,46]. Among these strategies, liposomal nanocarriers have been shown to be a promising topical and transdermal drug-delivery system in the therapy of local and distal tumors in numerous studies [47,48]. They can achieve much higher drug concentrations in the epidermis and dermis and lower systemic concentrations in a sustained pattern to enhance therapeutic efficacy and avoid systemic side effects when compared to conventional dosage forms.

## 3. Liposomes in Skin Delivery

Liposomes were introduced in 1965 by Bangham et al. as a model of membrane [49] and first suggested as topical drug delivery system in 1980 [50]. Liposomes are phospholipid vesicles comprising one or more concentric phospholipid bilayers (with or without additives) surrounding an inner aqueous compartment. In the lipid domain of the bilayer membrane, lipophilic drugs can be encapsulated, and in the aqueous core, hydrophilic drugs can be loaded. Due to their biphasic characteristics and diversity in design, composition, and construction, liposomes offer a dynamic and adaptable technology for enhancing drug delivery and targeting. Liposomes have similarity to native cell membrane phospholipid domains and have a ‘generally regarded as safe’ (GRAS) status in contrast to other carrier systems [51,52]. The liposome encapsulation efficiency of drugs depends on both the physicochemical properties of the drug, such as lipophilicity and the factors including liposomal bilayer composition and the method of preparation [51,52,53].

Liposomes can be generally classified according to their particle size, as small unilamellar vesicles (SUVs, 20–100 nm), large unilamellar vesicles (LUVs, 100–500 nm), and multilamellar vesicles (MLVs, 500–1000 nm). In general, the penetration of liposomes through the stratum corneum decreases with increasing diameters. The preferred liposome size for drug delivery is 50–500 nm in diameter [54].

Based on the components, liposomes can be classified into conventional liposomes and novel deformable/elastic liposomes which mainly include transfersomes, ethosomes, and invasomes. Conventional liposomes are composed of phospholipids with or without cholesterol. Since the first conventional liposome product as a topical formulation of econazole was introduced in the market in 1988 for dermatomycosis therapy, some other conventional liposomal products were clinically available for the topical delivery of antifungal, anti-inflammatory, and anticancer drugs [55,56]. Conventional liposomes were suggested mainly for topical drug delivery [57,58,59,60].

Transfersomes are ultradeformable vesicles consisting of a lipid bilayer with phospholipids and an edge activator (such as sodium cholate, Tween 80, span 80) which increase the bilayer elasticity and deformability of the vesicles [61,62]. The elastic vesicles applied non-occlusively on skin were assumed to have adequate driving force to penetrate through the SC under the hydration forces which are generated due to the hydration gradient across the epidermis [61]. The enhanced skin permeability of drugs by transfersomes were reported in numerous studies [62,63,64,65,66,67], including those for chemotherapeutics included in this review.

Ethosomes are composed of phospholipid and a high concentration (20–45%) of ethanol. The high ethanol content gives the phospholipid bilayer a fluid state and enhances the transdermal delivery for a range of drugs [68,69,70].

Invasomes are composed of phosphatidylcholine, ethanol, and a mixture of terpenes as penetration enhancers, which can also make the phospholipid bilayer fluid and flexible [71]. Drug deposition enhancement in skin was reported in some studies [72,73,74].

The great diversity of potential composition of liposomal vesicles and physicochemical characteristics such as particle size, charge, thermodynamic phase, and bilayer elasticity has significant effects on the interaction between vesicles and the skin, and hence on the effectiveness of these vesicles as topical and transdermal delivery systems. They offer a promising strategy for achieving the purpose of improved drug delivery through the skin [69,72,75].

Detailed reviews of the structures, production methods, characterization, properties, and potential skin permeation enhancement mechanisms of conventional and deformable liposomes can be found in [48,76,77,78].

## 4. Liposomal Nanocarriers in Skin Delivery of Chemotherapeutics for Cancer Therapy

Chemotherapy is the main treatment option for many aggressive and severe types of cancers. Currently, the routes of administration are mainly intravenous and oral. They cause very strong side effects due to their poor targeting and cytotoxicity to normal tissues. Liposomal nanocarriers have been extensively investigated as topical and transdermal drug delivery systems to enhance the therapeutic effect and avoid systemic side effects for numerous anticancer chemotherapeutics. The recent advances achieved by employing liposomal nanocarriers to enhance the skin delivery of clinically approved chemotherapeutic drugs aiming for optimal anticancer therapy are reviewed below.

### 4.1. Conventional Liposomes

Letrozole is a first-line drug in the treatment of estrogen-dependent breast cancer. It is a reversible inhibitor of the enzyme aromatase and blocks the production of estrogen. Letrozole is available as tablets for oral administration, which can significantly decrease the plasma levels of estrogen [79]. However, estrogen levels in the breast tissues can be significantly higher than in the plasma [79,80]. In addition, letrozole tablets induce systemic side effect such as nausea, vomiting, and hot flashes.

Letrozole has suitable physicochemical properties (log P_oct_ of 1.27, MW of 285.3, see Table 1) to be delivered through the skin. To achieve greater site-specificity and bioavailability, Maniyar et al. formulated Letrozole-loaded liposomes (size 284 nm, consisting of phospholipon 85^®^/cholesterol) dispersed in cream containing permeation enhancer for topical delivery to breast cancer [81]. In vitro experiments using MTT assay indicated that the optimized letrozole-loaded liposomal cream had superior anti-proliferative activity with lower viability in MDA-MB-231 breast cancer cells compared to the group treated with plain letrozole cream. An ex vivo study on rat abdominal skin showed the capacity of drug deposition in skin, increased permeation, and extended manner of drug release as compared to plain drug cream. An in vivo study in Wistar rats showed that the topical application of letrozole-loaded liposomal cream was superior to that of plain letrozole cream: about a four-fold increase in the maximum plasma concentration (C_max_), higher AUC values, and longer half-life (t_1/2_) of the drug. The authors explained that the permeation enhancer in the cream and the liposome caused a noteworthy change in skin fluidity that supported enhanced drug delivery. The study suggested that letrozole-loaded liposomal cream was effective in delivering the drug across the skin into the local tumor site and to the systemic circulation. The transdermal delivery of letrozole-loaded liposomal cream could potentially overcome the side effects caused by the oral administration as the drug is localized more in the targeted site than in systemic circulation.

Tamoxifen is an antagonist of the estrogen receptor and clinically used for the chemotherapy of estrogen receptor-positive breast cancer. It is available in the form of solution and tablets for oral administration. However, routinely taking tamoxifen orally can cause a range of side effects. The most concerning side effect is endometrial disease in postmenopausal women [82]. To overcome the side effects caused by oral administration, Lin et al. developed a transdermal tamoxifen-loaded liposome (consisting of DOPC/DLPC)-PEG-PEI complex (LPPC/TAM) with a particle size of 270 nm [83]. LPPC/TAM displayed dramatically increased (6.7 to 7.9-fold) cytotoxic activity in all breast cancer cells, especially in estrogen receptor-positive breast cancer cells, compared to the free drug and the blank carrier LPPC. It is postulated by the authors that LPPC is capable of delivering large quantities of encapsulated drugs across the cell membrane quickly for enhanced cytotoxic effects. The topical administration of LPPC/TAM achieved drug accumulation in the subcutaneous tumor site and inhibited about 86% of tumor growth in mice bearing a BT474 tumor, without injury of skin and any organs. The results indicated the potential feasibility of using liposomal complex formulations for the local delivery of tamoxifen in breast cancer therapy, to reduce the side effects of systemic administration.

### 4.2. Deformable/Elastic Liposomes

Bleomycin is an antitumor drug used in the treatment of non-melanoma skin cancer (NMSC). It is clinically available as powder for injection and can cause severe pulmonary toxicity [84]. To reduce systemic side effects, Hiruta et al. developed bleomycin-encapsulated deformable liposomal formulations incorporating a skin permeation enhancer, beta-sitosterol 3-β-D-glucoside (Sit-G), for topical application [85]. The deformable liposomes consist of EPC/Tween 80 or sodium cholate/Sit-G with a particle size of 145–158 nm. An in vivo skin deposition and permeation study on Wistar rats showed that the non-occlusive application of the formulations significantly increased distribution of BLM in the epidermis and dermis compared with aqueous bleomycin solution. No detectable serum concentration of BLM was observed from the formulations. Confocal laser scanning microscopy (CLSM) revealed that the optimized vesicles can deliver encapsulated hydrophilic fluorescent probes into full-thickness skin. The authors discussed that the penetration of developed lipid vesicles through skin was related to the deformability of the vesicle membrane and can pass through pores smaller than their own diameter, which was also reported previously by Cevc et al. in their study [86]. The preliminary study indicates the potential of the proposed deformable liposomal nanocarriers to deliver bleomycin for topical chemotherapy of NMSC to reduce systemic toxicity.

Docetaxel is used in the treatment of breast, ovarian, and non-small-cell lung cancers. It is given by IV infusion. Docetaxel has molecular weight of 807.9 and log P_oct_ of 4.1, which are not favorable for skin delivery. Qiu et al. developed an elastic liposomal system to enhance the skin permeation using docetaxel as a model drug [87]. The elastic liposomes consist of SPC/sodium cholate/ethanol and have a particle size of 43 nm after sonication and 197 nm before sonication. The effect of the developed formulations on the passive permeation of docetaxel across both rat and porcine skin was investigated in vitro. The results showed that the elastic liposomes enhanced the skin permeability of docetaxel compared to conventional liposomes. The combination of microneedle pretreatment and elastic liposomes on porcine skin in vitro demonstrated a significantly enhanced transdermal flux of docetaxel. These preliminary results suggested that elastic liposomes combined with microneedle pretreatment could be a useful method to increase skin permeation of drugs with high molecular weight and high lipophilicity.

**Figure 3:**
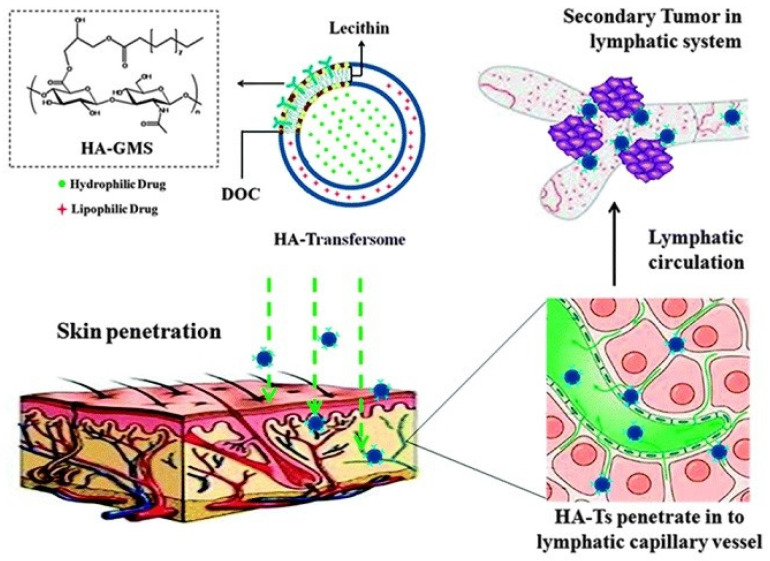
Schematic illustration of hyaluronic acid-modified transfersome (HA-Transfersome) for transdermal delivery of doxorubicin towards lymphatics. Reprinted with permission from [88].

Doxorubicin is a chemotherapy drug used to treat various types of cancer such as breast, bladder, lung cancer, and leukemia by injection. It has a molecular weight of 543.5 and log P_oct_ of 1.27. Kong et al. prepared a novel hyaluronic acid (HA)-modified transfersome loaded with doxorubicin to target lymphatic vessels for tumor metastasis therapy (see Figure 3) [88]. The transfersome consists of lecithin/sodium deoxycholate (DOC). HA with glycerol-α-monostearate modification (HA-GMS) was assembled onto the surface of the transfersome to form HA-Transfersome. The doxorubicin-loaded HA-Transfersome with a particle size of 251.4 nm enabled efficient drug penetration into the deep skin layer and targeted lymphatic drug delivery (with 10-fold stronger drug intensity in lymph nodes than doxorubicin-loaded transfersome without surface modification by HA-GMS) in Wistar rats, with limited accumulation of the drug in kidney and liver, which greatly reduced the systemic toxicity of doxorubicin. The developed transdermal nanocarriers have potential for the systemic treatment of metastatic cancers since lymphatic vessels are rich in the dermis.

As a potential strategy for killing the metastasized tumor cells through targeting dermal lymphatic vessels, doxorubicin-loaded transfersome integrated with a dissolving microneedle (MN) fabricated using hyaluronic acid was further developed by the same group [89]. In vivo studies revealed that the microneedles were able to efficiently insert into SD rat skin and release the doxorubicin-loaded transfersome in dermis via self-dissolution. No skin irritation was observed. The transfersome/microneedle complex could significantly promote the accumulation of doxorubicin in lymph nodes and increase its bioavailability.

5-Fluorouracil is a hydrophilic chemotherapeutic used for the treatment of different types of cancers when administered intravenously. A topical cream formation of the drug is also available for the treatment of skin cancer. 5-Fluorouracil has a molecular weight of 130.1 and log P_oct_ of −0.89. To achieve a synergistical therapeutic effect, Cosco et al. co-encapsulated hydrophilic 5-fluorouracil and lipophilic resveratrol in ultradeformable liposomes (Phospholipon 90G/cholesterol/sodium cholate) for the topical treatment of non-melanoma skin cancer [90]. Resveratrol is an antioxidant able to synergistically induce apoptosis in cancer cells. The in vitro anticancer activity of the developed ultradeformable liposomes was tested on human skin cancer cells through viability-, cell cycle- and apoptosis-analysis, and exhibited improved anticancer activity on skin cancer cells as compared to both the free drug form and the single entrapped agents. An in vitro permeation study on human epidermis showed that the encapsulation of 5-fluorouracil and resveratrol in ultradeformable liposomes led to a significant improvement of their percutaneous permeation with respect to the free drugs. The study also demonstrated that resveratrol was able to modulate the lipid bilayer fluidity of the liposomes and the release profile of 5-fluorouracil from the developed liposomes. The study suggested that ultradeformable liposomes might accumulate in deeper skin layers, thus generating a cutaneous depot from which 5-fluorouracil and resveratrol are gradually released to the target.

In another study, 5-fluorouracil-loaded transferosomes (phosphotidylcholine/Span80 or Tween80, 266.9 nm) dispersed in carbopol gel were evaluated for efficacy in the treatment of skin cancer [91]. An in vitro study using mice skin showed that the formulation enhanced the skin permeation and deposition of the drug compared to the marketed 5-fluorouracil cream. In in vivo efficacy studies, the mice treated with 5-fluorouracil-loaded transfersomal gel were observed with the maximum reduction in the skin tumor size compared to the marketed formulation after six weeks, indicating that a higher drug concentration in the tumor site was achieved by the transfersomal gel. No skin irritation was observed from the developed transfersomes.

Mitoxantrone is a chemotherapeutic for the treatment of cancers such as leukemia, non-Hodgkin lymphoma (NHL), and breast cancer. It is generally administered via the intravenous (i.v.) route and causes severe side effects such as myelo-suppression and cardiotoxicity [92,93]. Yu et al. loaded mitoxantrone in ethosome (soybean phospholipid/ethanol) dispersed in gel with a particle size of 78 nm for topical delivery to treat melanoma [94]. The formulation showed a much higher permeability of mitoxantrone across the rat skin in vitro than mitoxantrone aqueous solution. In a pharmacodynamic study on melanoma-bearing mice, mitoxantrone-loaded ethosomes exhibited an enhanced anti-melanoma effect compared to an MTO solution. The developed ethosome is a promising transdermal delivery system for melanoma therapy with the advantages of non-invasion and no significant side effects.

**Figure 4:**
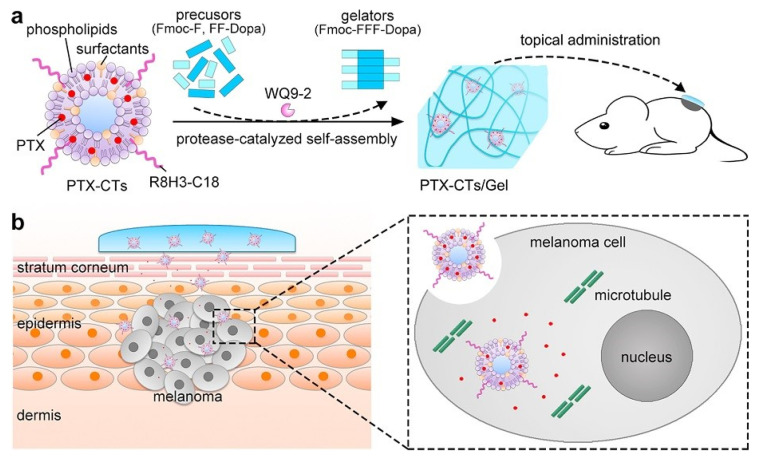
(**a**) Schematic illustration of preparation and application of PTX-CTs/Gel for topical drug delivery. (**b**) Schematic illustration of enhancement on the transdermal efficiency of PTX by the PTX-CTs/Gel for non-invasive chemotherapy of melanoma. Reprinted with permission from [96].

Paclitaxel is one of the most widely used and effective antineoplastic agents with a wide spectrum of antitumor activity. It is very lipophilic, with a log P_oct_ of 3.96 and molecular weight of 853.9. The formulation widely used in the clinical setting is a solubilized form of the drug in a 1:1 *v*/*v* mixture of Cremophor EL with dehydrated ethanol and diluted before IV administration. However, Cremophor EL can cause side effects such as hypersensitivity [95]. Jiang et al. developed paclitaxel (PTX)-loaded cell-penetrating-peptide (CPP)-modified transfersomes (PTX-CTs) dispersed in oligopeptide hydrogel (PTX-CTs/Gel) for topical melanoma treatment (Figure 4) [96]. The transfersomes are composed of soybean phospholipid/Tween 80/sodium deoxycholate with a particle size of 75 nm. The cell-penetrating peptide (CPP) is modified on the surface of transfersomes to further improve both the skin and tumor penetration capability of the formulation. After application on the upper skin of the B16F10 melanoma-bearing mice, PTX-CTs/Gel promoted PTX to efficiently permeate the skin and deliver into the melanoma cells to induce cell death. It also exhibited the superior capacity of tumor penetration and intracellular delivery. The tumor growth was significantly suppressed after topical treatment with PTX-CTs/Gel combined with the systemic administration of the commercial PTX preparation.

In another study, paclitaxel was formulated in transfersomes composed of soya phosphatidylcholine/span80 with particle size of 168 nm [97]. In vitro permeation and deposition studies on rat skin showed a 10.8-fold enhanced transdermal flux and 15.0-fold enhanced drug deposition in comparison to a drug solution prepared in propylene glycol (PG)/ethanol (7:3). The in vitro hemolytic toxicity study using a red blood cell lysis assay indicated that the developed formulation induced only 11.2% hemolysis in comparison to the commercial formulation which induced 38%. The in vivo study on rabbits showed no skin irritation. The results of the study further indicated that the transfersome approach is a promising option for skin delivery of paclitaxel.

Raloxifene is used for the treatment of invasive breast cancer. The oral administration shows very poor bioavailability of the drug. To overcome this issue, raloxifene-loaded transfersome was developed for transdermal delivery [98]. The raloxifene-loaded transfersomes were prepared using Phospholipon 90G/sodium deoxycholate with particle size of 134 nm. An in vitro permeation study on rat skin showed that the transdermal flux was significantly enhanced compared with drug-loaded conventional liposomes or drug solution in ethanolic phosphate-buffered saline. The study also confirmed that the skin treated with transfersomes was more fluid compared with untreated skin. The preliminary results indicated the potential of the transfersomal formulation of raloxifene via skin delivery could be an alternative to oral delivery for breast cancer treatment.

4-OH Tamoxifen is the active metabolite of tamoxifen. Sundralingam et al. developed 4-OH tamoxifen-loaded transfersome (soy phosphatidylcholine/sodium taurocholate) with/without emu oil for transdermal delivery [99]. The particle size of the developed transfersomes was 122–250 nm [100]. The topical application of the formulation gave comparative results with oral tamoxifen in the reduction in tumor size in breast cancer-bearing mice. However, the transfersome formulations resulted in a significantly lower plasma concentration of 4-OH tamoxifen compared to the oral administration of tamoxifen, which indicated that the transdermal route could achieve an effective drug concentration in the tumor site and avoid systemic circulation and thus reduce the distribution of the drug to tissues that are susceptible to tamoxifen or 4-OH tamoxifen-induced toxicity. No skin irritancy was found in the animals studied.

**Figure 5:**
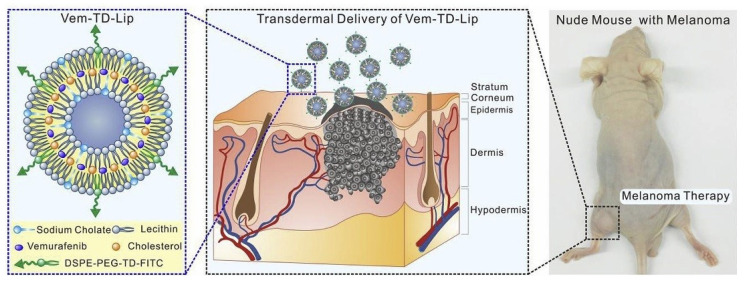
Schematic of transdermal Vem-TD-Lip in in vivo study on nude mouse with melanoma. Reprinted with permission from[103].

Vemurafenib is a chemotherapeutic drug for the treatment of melanoma. It is available as tablets for oral administration. Clinical studies indicate that the oral formulation of vemurafenib can produce hepatic and renal toxicity [101,102]. Zou et al. developed a vemurafenib-loaded TD1 peptide-modified liposome (Vem-TD-Lip) for the targeted inhibition of subcutaneous melanoma via transdermal delivery [103]. The deformable liposome is composed of lecithin/cholesterol/sodium cholate and has a particle size of 106 nm. An in vitro permeation study on the abdominal skin of Wistar rats showed that the conjugation of the TD-1 peptide on the surface of vemurafenib-loaded liposomes significantly promoted the skin penetration of vemurafenib, compared to the bare vemurafenib. An in vivo study on nude mice (see Figure 5) demonstrated that the topical administration of Vem-TD-Lip significantly enhanced the antitumor efficacy against melanoma by targeting melanoma cells harboring BRAF mutations and reduced the side effects to major organs compared with both oral and intravenous administration, indicating a higher drug concentration achieved at the melanoma site via the topical route. The promising results indicate that the TD1 peptide-modified liposomal system is a promising approach for improving transdermal efficacy with good biocompatibility for melanoma therapy.

Vismodegib is used for the prevention and treatment of skin cancer. It is available as a capsule for oral administration, but with poor bioavailability. Salen et al. prepared vismodegib-loaded invasomes (phospholipon 90G/cholesterol/cineole/ethanol, 188 nm) dispersed in Carbopol gel for skin cancer treatment via skin delivery [104]. The developed formulation demonstrated sustained drug release in the assays using a dialysis bag as a diffusion membrane. An in vivo study on malignant skin tumor (pappillomas)-bearing rats showed that vismodegib-loaded invasomes had 3.59-times-higher bioavailability (with lower plasma C_max_ and significantly higher AUC) and an excellent antitumor effect as compared to oral vismodegib, and no signs of skin toxicity. The study indicated the developed invasomes are potentially efficient carriers for delivering VSD via the transdermal route to treat skin cancer systemically.

The studies discussed above evidenced the efficacy of conventional liposomes and deformable liposomes (transfersome, ethosome, and invasome) on enhancing topical delivery of the chemotherapeutics into the skin, and to the systemic circulation to treat superficial cancers such as skin cancer and breast cancer, tumor metastasis, and distal cancers. The possible penetration enhancement mechanism by liposomes were also postulated by the studies in this review. However, no clear evidence-based understanding was established about the underlying enhancement mechanisms. The previous studies suggested that liposomes enhance drug penetration by either fusing with the SC lipids or by disturbing the intercorneocyte lipid organization [45,105,106]. Cevc et al. claimed that transfersomes could penetrate intact skin upon topical application owing to the transdermal osmotic gradients and hydration force [61], and that the penetration of transfersomes through the skin was related to the deformability of the vesicle membrane which enables them pass through pores smaller than their own diameter [86]. Jain proposed that ethanol in ethosomes decreased the phase-transition temperature of the SC lipid bilayers, leading to phase separation and improving ethosomes across the gaps smaller than the ethosomes themselves, i.e., the ethosomes could likely reach the deep layers of the skin [107]. The possible mechanisms of liposome-skin actions were also proposed by EI Maghraby et al. based on results of huge number of studies [18].

Table 1 summarizes the experimental parameters and drug physicochemical properties used in the studies of this review. It should be noted that the liposome size in these studies is between 43 and 663.8 nm (mostly in the range of 100–300 nm), indicating the key role of the proper liposome size in the enhancement of drug skin delivery. From Table 1, we can see that most chemotherapeutics discussed in this review do not have favorable physicochemical properties for skin delivery, with log P_oct_ values outside of the optimal range of 1–3, and a MW higher than 400. However, the promising results from the studies demonstrated the successful applications of liposomal systems on enhancing the topical and transdermal delivery of the molecules with a wide range of physicochemical properties.

## 5. Conclusions and Perspective

The skin delivery of chemotherapeutic drugs has been proved to be a promising alternative to the conventional oral and parenteral routes for cancer therapy, to enhance the therapeutic efficacy and reduce systemic side effects by achieving a high drug concentration at the target tumor site. The main challenge of the transdermal approach is the SC, a strong barrier which limits the permeation of most drugs to achieve effective local or plasma concentration. Liposomal nanotechnology provides a promising strategy to enhance and facilitate the skin permeation of chemotherapeutic drugs with diverse physicochemical properties. Furthermore, the carrier system can load and deliver two or more drugs to obtain a synergistical therapeutic effect. The preliminary studies summarized in this review demonstrated promising translational applications of the liposomal transdermal delivery systems in targeting local skin cancer, breast cancer, and tumor metastasis and delivering the drug to systemic circulation to treat distal cancers with sustained drug release, enhanced therapeutic efficacy, and reduced systemic side effects.

Despite the advances and positive implications of liposomal transdermal systems, systematic investigations on the effects of liposomal composition, surface charge, elasticity, and additives such as edge activator on the loading and permeation of drugs are missing. Although the possible permeation enhancement mechanisms by liposomes are proposed to be the vesicle fusion with the SC and the intact vesicle permeation (in the case of transfersomes), it is not clear how the mechanisms differ for the drugs with different physicochemical properties. As shown in our own study [73], the same liposomes can significantly improve the delivery of hydrophilic drug (carboxyfluorescein) into the deep layer of the skin, but they can only deliver hydrophobic drugs (e.g., temoporfin) to the superficial skin layer. The drug release mechanisms from liposomes to the skin layers are not well understood. Fundamental studies need to be carried out to unravel the permeation enhancement mechanisms of liposomal carriers for drugs with a wide range of physicochemical properties such as lipophilicity and molecular weight. The rational design and optimization of the liposomal formulations need to be established for the chemotherapeutics for the maximum efficacy and safety in the early stage, followed by the clinical translation of the transdermal therapeutic systems.

**Table 1:** Brief summary of liposomal nanocarrier composition and characteristics, physicochemical properties of the drug, and experimental design.

Liposome Components	Anticancer Drug	log P_oct_ ^a^	MW	Physicochemical Characteristics of the Drug-Loaded Liposomes	Main Assays	Ref.
*Conventional liposomes*						
phospholipon 85^®^/cholesterol	Letrozole	1.27	285.3	Size: 284 nm Zeta potential: −54.61 mV	In vitro MTT assay. Ex vivo drug permeation study with rat skin. In vivo bioavailability study in rats.	[81]
DOPC/DLPC	Tamoxifen	5.93 (predicted)	371.5	Size: <270 nm Zeta potential: ~40 mV	Cytotoxic assays against breast cancer cells. In vivo antitumor assay in mice bearing BT474 xenografts.	[83]
*Deformable liposomes*						
EPC/Tween 80 or sodium cholate/sit-G	bleomycin	−0.41	1415	Size: 145–158 nm Zeta potential: −3.48—30.7 mV	In vivo drug permeation and deposition study on rat skin. In vivo distribution of fluorescent-labeled vesicles by CLSM.	[85]
SPC/sodium cholate/ethanol	Docetaxel	4.10	807.9	Size: 43–197 nm	In vitro drug permeation study with deformable liposomes on rat and porcine skin. In vitro drug permeation study with microneedle pretreatment and deformable liposome on porcine skin.	[87]
lecithin/sodium deoxycholate/HA-GMS	Doxorubicin	1.27	543.5	Size: 251.4 nm	In vitro drug permeation study on rat skin. In vivo drug distribution study in rats.	[88]
lecithin/sodium deoxycholate	Doxorubicin	1.27	543.5	Size: 240.6 nm	In vitro assays of microneedle insertion in rat skin. In vivo transdermal study of transfersome/microneedle complex on rats. Skin irritation assay.	[89]
Phospholipon 90G/cholesterol/sodium cholate	5-Fluorouracil (5-Fu)	−0.89	130.1	Size: 316.6–663.8 nm Zeta potential: −25.5–−32.8 mV	In vitro assays against human skin cancer cells. In vitro permeation study on human epidermis.	[90]
phosphotidylcholine/Span 80 or Tween 80	5-Fu			Size: 195–330 nm	In vitro drug permeation and deposition study on mice skin. In vivo efficacy assay to reduce tumor size in mice. Skin irritation assay on mice.	[91]
soybean phospholipid/ethanol	Mitoxantrone	0.91	517.4	Size: 78 nm Zeta potential: −55 mV	In vitro permeation study on rat skin. In vivo anti-melanoma assay B16 melanoma-bearing mice.	[94]
soybean phospholipid/Tween 80/sodium deoxycholate	Paclitaxel	3.96	853.9	Size: 75 nm Zeta potential: +21 mV	In vitro permeation study on mice skin. In vivo anti-melanoma assay on B16F10 melanoma-bearing mice.	[96]
soya phosphatidylcholine/ span80	Paclitaxel	3.96	853.9	Size: 168 nm	In vitro permeation and deposition studies on rat skin. In vitro hemolytic toxicity assay. In vivo skin irritation assay on rabbit.	[97]
Phospholipon 90G/sodium deoxycholate	Raloxifene	5.45	473.6	Size: 134 nm Zeta potential: slightly negative	In vitro permeation study on rat skin. In vitro study of distribution of fluorescent-labeled vesicles on rat skin by CLSM.	[98]
soy phosphatidylcholine/ sodium taurocholate	4-OH tamoxifen	5.44 (predicted)	387.5	Size: 122–250 nm Zeta potential: −4.75 mV	Skin irritancy assay on mice. In vivo antitumor study on breast cancer-bearing mice. Quantification of drug plasma concentration in mice.	[99]
lecithin/cholesterol/ sodium cholate	Vemurafenib	5.10	489.9	Size: 106 nm Zeta potential: −4.75 mV	Cytotoxic assays against melanoma cells. In vitro permeation study on rat skin. In vivo antitumor study on melanoma-bearing mice.	[103]
phospholipon 90G/cholesterol/cineole/ ethanol	Vismodegib	2.70	421.3	Size: 188 nm Zeta potential: −4.75 mV	In vitro drug release assay. In vivo permeation and bioavailability study on rats. In vivo deposition study in rat skin. In vivo antitumor activity on skin tumor bearing rat.	[104]

^a^: Partition coefficient in n-octanol/water; all are experimental values unless noted otherwise. Taken from Drugbank [108].

## Data Availability

The data produced in this study are included within the paper.
